# The prevalence of denture related mucosa lesions among patients managed in a Nigerian teaching hospital

**DOI:** 10.11604/pamj.2020.37.358.22194

**Published:** 2020-12-21

**Authors:** Tunde Joshua Ogunrinde, Olalekan Fatai Olawale

**Affiliations:** 1 Department of Restorative Dentistry, University of Ibadan and University College Hospital, Ibadan, Nigeria

**Keywords:** Prevalence, denture, mucosa lesions

## Abstract

**Introduction:**

the prevalence of denture related mucosa lesions (DRML) varies across different countries and a recent study reported an increasing trend in its prevalence. The objective of this study was to determine the prevalence of DRML and factors related to the lesions among denture wearers seen in a Nigerian teaching hospital.

**Methods:**

interviewer’s administered questionnaire was used to obtain information from consecutive patients that had used removable denture for at least six months and consented to participate. Data related to gender, age, types of denture and presence of denture induced oral lesions were obtained, entered into a computer and analyzed using IBM SPSS Version 20. Descriptive statistics were expressed as frequency and percentages. Fisher’s exact test was performed for discrete variables. A P-value less than 0.05 was regarded as statistically significant.

**Results:**

a total of 104 respondents participated in the study and 14 had DRML giving a prevalence of 13.5%. The majority, 11 out of the 14 (78.57%) presented with mucosa ulceration, while 8 (57.14%) out of the 14 cases of DRML were caused by over extension of the denture flanges. There was no statistically significant relationship between daily removal of denture fore going to bed to sleep at night and DRML (p=0.776).

**Conclusion:**

the prevalence of denture related mucosal lesion was 13.3% and the major cause was over extension of denture flange. There is need to emphasize adherence to review appointments for early detection and correction of denture instability and over extension of denture flange to prevent DRML.

## Introduction

Worldwide, the prevalence of edentulism is high and varies considerably between countries (19% in Italy, 46% in the United Kingdom, and 58% in Canada) [1]. In Nigeria, a prevalence of 33.6% among the Hausa community dwelling in the South West region was reported by Oremosu and Uti [2] while a prevalence of 43.6% among the elderly in the South South region was reported by Bashiru and Ovenashia [3]. The use of denture has been shown to remediate the negative consequences of edentulism such as problems with speech, mastication and aesthetics [4] and the most frequently employed prosthesis is removable denture, especially in developing nations [5]. Previous studies [6,7] showed that the wearing of removable dentures could lead to oral mucosal lesions. The lesion may result from mechanical denture trauma, traumatic occlusion, chronic irritation from loose denture, reaction to components of denture base material, and acute or chronic reactions to microbial denture plaque [8]. These lesions can present as localized or generalized areas of hyperaemia, traumatic ulcer, denture induced mucosa hyperplasia, flabby ridges, denture stomatitis and less commonly oral carcinomas [9].

The prevalence of denture induced oral lesions varies across different countries and ranges from 10.8% to 62% [10,11]. A prevalence of 10.8% was reported by Feng *et al*. [10] among Chinese population, da Silva *et al*. [12] reported a prevalence of 50% among Brazilian farmers wearing dentures while Gaur *et al*. [13] reported a prevalence of 59.4% among India population. In Nigeria, a community-based study [14] revealed a prevalence of 3.9% denture related mucosa lesions. Denture stomatitis was reported as the most common denture related mucosa lesion by several studies [11-13]; Sadig *et al*. [11] reported the prevalence of denture stomatitis to be 62%, da Silva *et al*. [12] 48.2% and Gaur *et al*. [13] 59.25%. DRMLs such as traumatic ulcers (3.6%) [12], epulisfissuratum (18.5%) [13], angular cheilitis (5.4%) [12] and papillary hyperplasia (7.4%) [13] occurred less commonly. Denture induced oral mucosal lesions can cause great discomfort to the patient, affect the stability and retention of the denture and discourage regular use of denture [15]. This can impair oral functions such as speech, mastication and aesthetics [4]. In addition, denture induced chronic injury of the oral mucosa may predispose to oral carcinoma, although this is very rare [9]. Coelho *et al*. [16] reported an increasing trend in the prevalence of denture induced oral mucosa lesion. But to the best of our knowledge there was no Nigerian clinic-based study reporting the prevalence and the factors related to the incidence of DRMLs at the commencement of this study. A knowledge of the prevalence of DRMLs and associated factors will help in formulating appropriate preventive measure to reduce the prevalence. The purpose of this study was therefore to determine the prevalence of denture related mucosal lesions and factors related to the occurrence of the lesions in patients treated with removable dentures at the dental centre of a Nigerian teaching hospital.

## Methods

This was a cross-sectional study among removable denture wearers. A total of one hundred and four removable denture wearers were interviewed over a period of twelve months. Only patients that were willing to participate and had used the removable denture for at least six months were included in the study. After informed consent was obtained, data related to gender, age, type of denture, length of denture use, hygiene care, nocturnal denture wear, and presence of denture induced oral lesion were obtained using an interviewer´s administered questionnaire. The questionnaire was administered by one of the investigators. Confidentiality was ensured by not writing the name of the participants in the data form. Examination of each patient´s mouth was done on a dental chair by using a mouth mirror with gloved hand. Diagnosis of denture induced oral mucosa lesion was made based on the clinical appearance of the oral mucosa; denture stomatitis based on inflamed palatal mucosa, and ulceration was diagnosed based on presence of laceration or tearing on the oral mucosa in relation to denture. Patients with nodular mucosal swelling were referred to oral surgery clinic for excisional biopsy and the biopsies were sent to oral pathology laboratory for histological confirmation of mucosal hyperplasia. Also, the patients´ dentures were examined, and according to the quantity of plaque on the denture base, patients were divided into two groups by using Budtz-Jorgensen´s index [17] (modified) of denture cleanliness. Dentures were assessed clean when none or only few spots of plaque were present, and dirty when an extended part of the denture base (about half or more) was covered by plaque. Data collected were imputed into a personal micro-computer and analyzed using SPSS (version 20, IBM). Descriptive statistics were expressed as frequency and percentages. The relationship between dependent (DRML) and independent variables (type, cleanliness, overnight wearing and duration of denture use) were assessed using Fisher´s exact test. The level of significance was set at p <0.05. Ethical approval for the study was provided by the institutional health research ethical committee (UI/EC/19/0492).

## Results

A total of one hundred and four respondents participated in the study. The participants´ age ranged from 12 to 84 years with a mean of 53.94±17.76 years. Sixty-one (58.7%) of the respondents were females and majority 48 (46.2%) were above 60 years (Table 1). Table 2 shows that sixty-one (58.7%) of the respondents were using upper removable partial dentures (URPD) only, while 15 (14.4%) were using lower removable partial dentures (LRPD). Six patients (5.8%) came with upper or lower or upper and lower complete dentures (LCD). The majority 43 (41.3%) of the participants had used the same dentures for less than five years while 26 (25.0%) had used the same denture for more than 15 years. The majority 54 (51.9%) of the respondents remove their dentures at night every day before sleeping while 26 (25.0%) never removed their dentures. The majority 88 (84.6%) had nil or mild accumulation of plaque on their dentures (dentures assessed clean) while 16 (15.4%) had moderate to gross accumulation of plaque on his/her dentures (denture assessed dirty). Fourteen (13.5%) participants had oral mucosa lesions as a result of denture wearing. The site most commonly affected was the sulcus where 7 (50.0%) out of the 14 lesions occurred (5 (35.7%) occurred in the upper labial or buccal sulcus and 2 (14.3%) in the lower labial or buccal sulcus). The site least affected was the palatal area where 1 (7.1) out of the 14 cases of DRML was located. Mucosa ulceration was the most common lesion 11(78.6%) seen, and the least was papillary swelling 1 (7.1%). The most common cause of lesions was over extension of the denture flange affecting 8 (57.1%) of the participants, followed by inadequate relief over bony area affecting 4 (28.6%) individuals (Table 3).

**Table 1:** socio-demographic characteristic of the participants

Variable	n	%
**Sex**		
Male	43	41.3
Female	61	58.7
**Age group**		
≤20	3	2.9
21-40	24	23.0
41-60	29	27.9
≥60	48	46.2

**Table 2:** characteristics of dentures used by the respondents

Characteristics of dentures	n	%
**Types of denture**		
Upper removable partial dentures (URPD)	61	58.7
Lower removable partial dentures (LRPD)	15	14.4
Upper and lower removable partial denture	22	21.1
Upper complete denture	2	1.9
Lower complete denture	1	1.0.
Upper and lower complete denture	1	1.0.
UCD and LRPD	2	1.9
**Duration of usage of denture**		
<5 years	43	41.3
5-10 years	24	23.1
11-15 years	11	10.6
>15 years	26	25.0
**How often do you remove denture at night?**		
Everyday	54	51.9
Sometimes	14	13.5
Rarely	10	9.6
Never	26	25.0
**Denture cleanliness**		
Clean	88	84.6
Dirty	16	15.4
Total	104	100.0

**Table 3:** distribution of sites, forms and causes of denture induced oral lesions

Sites, forms and causes of lesions	n	%
**Any lesion**		
No	90	86.5
Yes	14	13.5
**Sites of lesions**		
Upper labial/buccal sulcus	5	35.7
Upper labial alveolus	2	14.3
Lower labial/buccal sulcus	2	14.3
Lower lingual alveolus	4	28.6
Rugae area of palate	1	7.1
**Forms of lesions**		
Hyperplasia/Epulisfissuratum	2	14.3
Papillary hyperplasia	1	7.1
Ulceration/redness	11	78.6
**Causes of lesions**		
Inadequate relief	4	28.6
Instability	2	14.3
Over extension	8	57.1
Total	14	100.0

There was no statistically significant relationship between daily removal of denture before going to bed to sleep at night and DRML (p=0.776), types of denture and DRML (p=0.269) and presence of plaque on the denture and DRML (p=0.729) (Table 4). Table 4 also shows that 5 (71.4%) out of 7 lesions that occurred in the sulcus involved patients with removable partial denture (RPD) while 7 (100%) located in the alveolus or palate were seen in patient with RPD. However, there was no statistically significant relationship between type of denture and site of lesion (p=0.231). Also, one (33.3%) out of 3 cases of mucosa or papillary hyperplasia involved RPD while the 11 (100%) cases of mucosa ulceration were seen in RPD and the relationship between the form of lesions and type of dentures was statistically significant (p=0.033). In addition, 9 (81.8%) out of the 11 cases of denture related mucosa ulcerations involved patients with denture that were in use for up to 5 years while 1 (33.3%) of the cases of hyperplasia involved dentures that were in use for up to 5 years. However, the relationship between the duration of denture use and form of lesions was not statistically significant (p=0.176). Also, 5 (71.4%) out of the 7 cases of DRML located in the sulcus were seen in patients that had used their dentures for up to 5 years´ duration while 2 (28.6%) cases were seen in denture wearers of more than 5 years´ duration. However, the relationship between duration of denture use and site of lesions was not statistically significant (p=1.000).

**Table 4:** relationship between sleeping with denture overnight, type of denture, denture cleanliness, duration of use of denture and denture related mucosa lesion (DRML)

Variables		Presence of denture related mucosa lesions				P-value
Denture removal at night		Yes n (%)		No n (%)		
Every day		8 (57.1)		46 (51.1)		
Not every day		6 (42.9)		44 (48.9)		0.776
**Types of denture**						
RPD		12 (85.7)		86 (95.6)		
CD		2 (15.4)		4 (4.4)		0.269
**Denture cleanliness**						
Clean		12 (85.7)		76 (84.4)		
Dirty		2 (14.3)		14 (15.6)		1.000
**Types of denture**	**Site of lesion**		**P-value**	**Form of lesion**		
	n (%)	n (%)		n (%)	n (%)	
RPD	5 (71.4)	7 (100)	0.231	1 (33.3)	11 (100)	0.033
CD	2 (28.6)	-		2 (66.7)	-	
**Duration of use**						
≤5 years	5 (71.4)	5 (71.4)	1.00	1 (33.3)	9 (81.8)	
>5 year	2 (28.6)	2 (28.6)		2 (66.7)	2 (18.2)	0.176

RPD = Removable partial denture; CD = Complete denture

## Discussion

In this study 58.6% of denture wearers that participated were females and this is in agreement with a previous study by Arigbede *et al*. [18]. The female predominance could be attributed to the facts that females are more conscious of aesthetics compared to males, so they readily present in the clinic for teeth replacement to improve their dental and facial appearance. The majority of the participants (46.2%) were aged 60 and above and this could be attributed to the fact that edentulism is commoner among the elderly group which is in agreement with the report of a previous study [19]. The prevalence of denture related oral mucosa lesions in this study was 13.5%, this is higher than 3.9% reported by Taiwo *et al*. [14] but lower than 59.5% prevalence reported by Gaur *et al*. [13] 45.6% by Pavicic *et al*. [20], 20.5% prevalence reported by Mubarak *et al*. [7]. The difference in prevalence could be explained by variations in characteristics of the population studied. Several studies [7,20,21] reported that the prevalence of denture induced oral lesion is higher among complete denture wearers than removable partial denture patients. Jainkittivong *et al*. [21] reported a prevalence of 46.1% in complete denture wearers against 43.6% in partial denture patients, while Pavicic *et al*. [20] reported a prevalence of 46.3% among complete denture wearers as against 40.8% among partial denture wearers. In this study the proportion of complete denture wearers was very low (6 out of 104 patients) although the prevalence of DRML was higher (33.3%) in the complete denture wearers than the partial denture wearers (12.2%). The lower prevalence reported by Taiwo *et al*. [14] could be because the study was community based in which majority were not denture wearers.

In this study, traumatic ulcer was the most common form of denture induce lesion 11(78.6%), followed by mucosa hyperplasia 2 (14.3%). This is in contrast to previous studies [11,13,21] that reported denture stomatitis as the most frequent DRML. Pavicic *et al*. [19] reported denture stomatitis as the most frequent followed by angular cheilitis. Gaur *et al*. [13] reported denture stomatitis as the most common followed by epulisfissuratum. The disparity could also be due to difference in the study population and the major cause of the mucosa lesion; this study assessed both partial and complete denture wearers with predominant removable partial denture wearers. In addition, it assessed the adult, middle age and elderly with the elderly constituting less than 50%. Denture stomatitis is more common among complete denture wearers and the elderly [13]. Our finding showed over extension of denture flanges to be the most common (57.1%) causes of denture induce oral lesion. This is in contrast to infection by *Candida albicans* being reported as a major cause by previous studies [6,13]. The most frequent site for denture induced oral lesions in our study was the sulcus (50.0%) followed by the alveolus. This is in contrast to report of previous study which reported the tongue and the palate [20] as the most frequent site affected by DRML. The reason for this finding in our study could be improper denture fabrication with over extended flange and inadequate relief over bony prominence or undercut ridge. Gaur *et al*. [13] stated that oral mucosal lesions can result from poorly adapted dentures, improper use and mechanical trauma from ill-fitting or over extended denture.

There is no significant relationship between not wearing the denture to bed at night and the development of DRML, although the group that sometimes remove their denture before bedtime had the highest (35.7%) incidence of DRMLs in this study. This is in contrast to the previous study that shows higher prevalence among those that wear denture to bed [11]. The higher prevalence of DRML among those that frequently remove their denture at night in this study may also be due to the inadequate relief of denture flange over undercut edentulous ridge which could be traumatic during insertion and withdrawal of denture. In this study a statistically significant relationship was established between the form of lesions and type of dentures with mucosa ulceration affecting individuals with RPD more than complete denture wearers, and hyperplasia associated more with complete dentures. This is in concordance with previous studies that stated that fibrous hyperplasia is more associated with complete denture and usually result from chronic irritation of the tissue in contact with ill-fitting denture border [12,13]. Stober *et al*. [22] reported that prevalence of DRMLs increased with the period of use of the same denture. The contrast is the case in this study, the frequency of DRMLs reduced among individuals that have used the same denture for a longer period. The reason for the observation by Stober *et al*. [22] was that the deterioration of the denture with time of use possibly increased the chance of developing DRMLs. The limitation of this study is the few number of patients with DRMLs which necessitated the categorisation of the patients into two: complete and partial denture wearers, patients with hyperplasia and ulceration, lesions located in the sulcus and the alveolus. This also dictated the use of Fisher´s exact test for analysis. We suggest a larger community based or multi-centre study to estimate more representative percentage of denture related mucosa lesions.

## Conclusion

The prevalence of denture related mucosal lesion is 13.5% in this study and the major cause was over extension of denture flange. There is need to emphasize proper denture fabrication to prevent DRMLs and adherent to review appointments for early detection of denture instability and over extension of denture flange.

### What is known about this topic

- Denture related mucosa lesion (DRML) has been studied and found to show varying prevalence across different countries;
- DRML was more common among complete denture wearer and in elderly;
- Denture stomatitis was found to be the most common form of DRML.

### What this study adds

- The causes and factors related to the incidence of denture related mucosa lesion (DRML) was studied for the first time in Nigeria and we found that ulceration was the major cause of DRML especially among RPD wearers;
- There was a statistically significant relationship between forms of lesion and types of denture. Mucosa ulceration and epulisfissuratum occurred more commonly among removable partial denture and complete denture wearers respectively;
- There was no statistically significant relationship between the duration of use of denture and prevalence of DRML.
